# Low radiographic muscle density is associated with lower overall and disease-free survival in early-stage colorectal cancer patients

**DOI:** 10.1007/s00432-018-2736-z

**Published:** 2018-08-17

**Authors:** Harm van Baar, S. Beijer, M. J. L. Bours, M. P. Weijenberg, M. van Zutphen, F. J. B. van Duijnhoven, G. D. Slooter, J. F. M. Pruijt, J. J. Dronkers, A. Haringhuizen, E. J. Spillenaar Bilgen, B. M. E. Hansson, J. H. W. de Wilt, E. Kampman, R. M. Winkels

**Affiliations:** 1 Division of Human Nutrition, Wageningen University and Research, PO Box 17, 6700 AA Wageningen, The Netherlands; 2 Netherlands Comprehensive Cancer Organisation (IKNL), Utrecht, The Netherlands; 3 Department of Epidemiology, GROW School for Oncology and Developmental Biology, Maastricht University, Maastricht, The Netherlands; 4 Department of Surgery, Maxima Medical Centre, Veldhoven, The Netherlands; 5 Department of Medical Oncology, Jeroen Bosch Hospital, ‘s Hertogenbosch, The Netherlands; 6 Department of Physiotherapy, Gelderse Vallei Hospital, Ede, The Netherlands; 7 Department of Medical Oncology, Gelderse Vallei Hospital, Ede, The Netherlands; 8 Department of Surgery, Rijnstate Hospital, Arnhem, The Netherlands; 9 Department of Surgery, Canisius Wilhelmina Hospital, Nijmegen, The Netherlands; 10 Department of Surgery, Radboud Universitair Medisch Centrum, Nijmegen, The Netherlands; 11 Department of Public Health Sciences, Penn State College of Medicine, Penn State University, Hershey, PA USA

**Keywords:** Colorectal cancer, Skeletal muscle density, Mortality, Survival

## Abstract

**Background:**

In cancer patients with a poor prognosis, low skeletal muscle radiographic density is associated with higher mortality. Whether this association also holds for early-stage cancer is not very clear. We aimed to study the association between skeletal muscle density and overall mortality among early-stage (stage I–III) colorectal cancer (CRC) patients. Furthermore, we investigated the association between skeletal muscle density and both CRC-specific mortality and disease-free survival in a subset of the study population.

**Methods:**

Skeletal muscle density was assessed in 1681 early-stage CRC patients, diagnosed between 2006 and 2015, using pre-operative computed tomography images. Adjusted Cox proportional hazard models were used to evaluate the association between muscle density and overall mortality, CRC-specific mortality and disease-free survival.

**Results:**

The median follow-up time was 48 months (range 0–119 months). Low muscle density was detected in 39% of CRC patients. Low muscle density was significantly associated with higher mortality (low vs. normal: adjusted HR 1.91, 95% CI 1.53–2.38). After stratification for comorbidities, the association was highest in patients with ≥ 2 comorbidities (HR 2.11, 95% CI 1.55–2.87). Furthermore, low skeletal muscle density was significantly associated with poorer disease-free survival (HR 1.68, 95% CI 1.14–2.47), but not with CRC-specific mortality (HR 1.68, 95% CI 0.89–3.17) in a subset of the study population.

**Conclusion:**

In early-stage CRC patients, low muscle density was significantly associated with higher overall mortality, and worse disease-free survival.

## Introduction

In recent years, there has been a growing interest in the influence of skeletal muscle (radio-) density on cancer prognosis. Skeletal muscle density, measured by computed tomography (CT) and quantified in Hounsfield units (HU), reflects the lipid content of the muscle cells (Goodpaster et al. 2000); higher muscle lipid content in the muscle cells presents as lower skeletal muscle density (Goodpaster et al. 2000). Lower skeletal muscle density has been found to be associated with higher total adiposity (Goodpaster et al. 2001), obesity (Lee et al. 2005) and increasing age (Anderson et al. 2013). In a recent study, an association between low skeletal muscle density and pre-existing comorbidities was also reported suggesting a potential shared mechanism between fat infiltration in the muscle and comorbidities (Xiao et al. 2018).

Various studies have reported an association between low skeletal muscle density and higher mortality (Sjøblom et al. 2016; Fujiwara et al. 2015; Rier et al. 2017; Dijk et al. 2017; Blauwhoff-Buskermolen et al. 2016b; Ebadi et al. 2017; Antoun et al. 2013) in cancer patients. Until now, majority of the studies have been performed in late-stage (stage IV) cancer patients (Sjøblom et al. 2016; Rier et al. 2017; Blauwhoff-Buskermolen et al. 2016b) and/or in cancer types with poor prognosis (Fujiwara et al. 2015; Dijk et al. 2017; Ebadi et al. 2017). Three studies (van Vugt et al. 2018; McSorley et al. 2017; Kroenke et al. 2018) investigated the association between skeletal muscle density and mortality exclusively in early-stage (stage I–III) colorectal (CRC) patients, who have a much better prognosis than stage V patients [i.e., 5 year survival rate of > 70 vs < 15% in late-stage CRC (Siegel et al. 2017)], and results were not consistent. The first two studies were performed in European populations and did not show significant associations between skeletal muscle density and overall mortality (van Vugt et al. 2018; McSorley et al. 2017), CRC-specific mortality (McSorley et al. 2017) or disease-free survival (van Vugt et al. 2018) in early-stage CRC patients after adjusting for confounding factors. A caveat is that both studies used cut-off points for low muscle density, which were defined in a mixed group of cancer patients of various diagnosis, mostly with very poor prognosis (Martin et al. 2013). Those cut-off points may not be appropriate in an early-stage European CRC patient population. The third study, performed in the US (Kroenke et al. 2018) reported that lower skeletal muscle density was associated with higher overall and CRC-specific mortality. Given the inconsistency of findings so far and the challenges in the interpretation in a European population, we decided to study the association between skeletal muscle density and overall mortality in a large cohort of European early-stage CRC patients. Furthermore, we investigated the association between skeletal muscle density and CRC-specific mortality and disease-free survival in a subset of the study population.

## Materials and methods

### Subjects

Data from two ongoing prospective cohort studies (*n* = 1111) were combined with registry-based data from three hospitals (*n* = 1537). The prospective cohort studies, i.e., the COLON (Winkels et al. 2014) and EnCoRe (van Roekel et al. 2014) studies, started in 2010 and 2012, respectively. Both investigated the role of lifestyle in CRC patients; details have been described earlier (Winkels et al. 2014; van Roekel et al. 2014). CRC patients included in the COLON study participants were diagnosed between August 2010 and November 2015; CRC patients included in the EnCoRe study were diagnosed between April 2012 and November 2014. For the registry-based data, the Netherlands Cancer Registry was used to select all stage I–III CRC patients diagnosed between January 2007 and December 2010 in two hospitals and January 2007 and January 2013 in a third hospital.

For all cohorts, exclusion criteria were: missing data on height, weight, stage of disease or comorbidities; stage IV CRC; missing or unusable CT images (i.e., CT images of poor quality or scans where muscle tissue was partly cut-off). Furthermore, only pre-operative CT images assessed within 3 months of diagnosis were considered representative for skeletal muscle status at diagnosis.

The COLON study was approved by the Committee on Research involving Human Subjects, region Arnhem–Nijmegen, the Netherlands. The EnCoRe study was approved by the Medical Ethics Committee of the University Hospital Maastricht and Maastricht University, the Netherlands. All participants of the COLON and EnCoRe study provided written informed consent. According to the Central Committee on Research involving Human Subjects (CCMO), studies using the data of the Netherlands Cancer Registry do not require approval from an ethics committee in the Netherlands. The study was approved by the Privacy Review Board of the Netherlands Cancer Registry. In addition, the ethical committees of the three participating hospitals gave permission to use additional data from CT images.

### Body composition and anthropometry

Skeletal muscle cross-sectional area was assessed using pre-operative CT images at the level of the 3rd lumbar vertebrae. Skeletal muscle was quantified using Slice-O-Matic 5.0 (Tomovision, Montreal, Canada). Standard density thresholds were used to define skeletal muscle: − 29 to + 150 HU (Mitsiopoulos et al. 1998). Standardized procedures were followed by trained researchers to correctly identify and quantify the psoas, erector spinae, quadratus lumborum, transversus abdominis, external and internal obliques, and rectus abdominis. Skeletal muscle density was measured as the mean density (in HU) of the total skeletal muscle cross-sectional area. Furthermore, as potential confounders, visceral adipose tissue, subcutaneous adipose tissue and intermuscular adipose tissue were quantified using the following standard density thresholds: − 150 and − 50 HU for visceral adipose tissue; − 190 and − 30 HU for both subcutaneous and intermuscular adipose tissue. Total adipose tissue was calculated as the sum of visceral, subcutaneous and intermuscular adipose tissue.

The association between skeletal muscle density and overall mortality, CRC-specific mortality and disease-free survival were assessed using continuous and dichotomized (low vs normal skeletal muscle density) variables. For the identification of patients with low skeletal muscle density, gender- and body mass index (BMI)-specific cut-off values were determined using optimal stratification (Williams 2006), which is a statistical method previously used in comparable studies (Martin et al. 2013; Prado et al. 2008; Caan et al. 2017) using a macro in SAS (version 9.3; SAS Institute, Cary, NC, USA). We decided to create specific cut-off values for our population, as established cut-off values to identify and low skeletal muscle density had been determined within cancer populations with a much higher mortality rate (Martin et al. 2013) or in other continents (North America) (Kroenke et al. 2018) which might not be representative for a European cohort. As acknowledged by others (Blauwhoff-Buskermolen et al. 2016a; Daly et al. 2016), reference cut-off values for an European population are important because of possible differences in body composition and prevalence of obesity between European, US and other populations. This optimal stratification procedure, resulted in the following cut-off levels for skeletal muscle density: for men with a BMI < 25 kg/m^2^ the cut-off value for low skeletal muscle density was 36.4 HU, for men with a BMI ≥ 25 kg/m^2^ 31.6 HU; for women with a BMI < 25 kg/m^2^ 31.1 HU; for women with a BMI ≥ 25 kg/m^2^ 29.3 HU.

Height (m) and weight (kg) at diagnosis were self-reported within the COLON study; measured by trained research assistants within the EnCoRe study; and collected from medical records by the Netherlands Cancer Registry for the registry-based data.

### Medical history and mortality

Data on age, gender, type of cancer, stage of disease, comorbidities, and date of surgery were collected from medical records for the prospective cohort studies and from the Netherlands Cancer Registry for the registry-based data. Mortality data were retrieved from the Municipal Personal Records Database. Overall mortality was measured as number of months alive after the assessment date of the CT image until time of death or January 31, 2017. Patients who were alive on this date were censored. For participants of the COLON study (*n* = 715), recurrence data was retrieved in collaboration with the Netherlands Cancer Registry and cause of death was obtained by linkage with Statistics Netherlands (CBS). The International Classification of Diseases 10th Revision (ICD-10) was used to identify CRC-specific mortality (ICD-10 codes C18–C20). DFS was calculated as number of months between the assessment date of the CT image and either a recurrence, metastasis of disease, or death from any cause.

### Statistical analyses

Hazard ratios (HR) and corresponding 95% confidence intervals (CI) for overall mortality, CRC-specific mortality and disease-free survival were obtained using Cox proportional hazard analyses. Proportional hazard assumptions were tested by log–log curves, with no violations noted.

The model for overall mortality was tested for effect modification by gender, age and number of comorbidities (0, 1, ≥ 2), by calculating the *p* value for interaction. Of these variables, the number of comorbidities was the only variable that was identified as an effect modifier for skeletal muscle density. Thus, the models for skeletal muscle density and overall mortality were stratified for number of comorbidities. Since no difference was found between zero and one comorbidity, these two categories were combined into one category: the final model was stratified into two groups (i.e., 0–1 comorbidity vs ≥ 2 comorbidities). Data on cause of death, recurrence and metastasis, were only available for the COLON study. Due to the limited sample size of the COLON study population (*n* = 715), statistical power precluded the possibility to assess effect modification in the analyses for CRC-specific mortality and disease-free survival.

Based on existing literature, age, stage of disease and gender were selected as potential confounding variables and were included in the final multivariable Cox proportional hazard models. Other variables, i.e., BMI (continuous and categorical), study type (prospective or registry-based), tumor location (colon or rectal), treatment (chemotherapy yes or no; radiotherapy yes or no), number of comorbidities (0–1 or ≥ 2), skeletal muscle index (i.e., skeletal muscle mass corrected for height in meters squared), visceral adipose tissue, subcutaneous adipose tissue and total adipose tissue were included in the final model if they changed the HR for mortality with 10% or more when the variable was individually added to the model. Based on these criteria, only age, gender and stage were included in the final adjusted models.

As a sensitivity analyses, we repeated all analyses, excluding patients who died or had a recurrence or death within 1 year of follow-up.

Level of significance was set at 0.05. Analyses were performed using IBM SPSS 23.0 (SPSS Inc., Chicago, IL, USA).

## Results

**Fig. 1:**
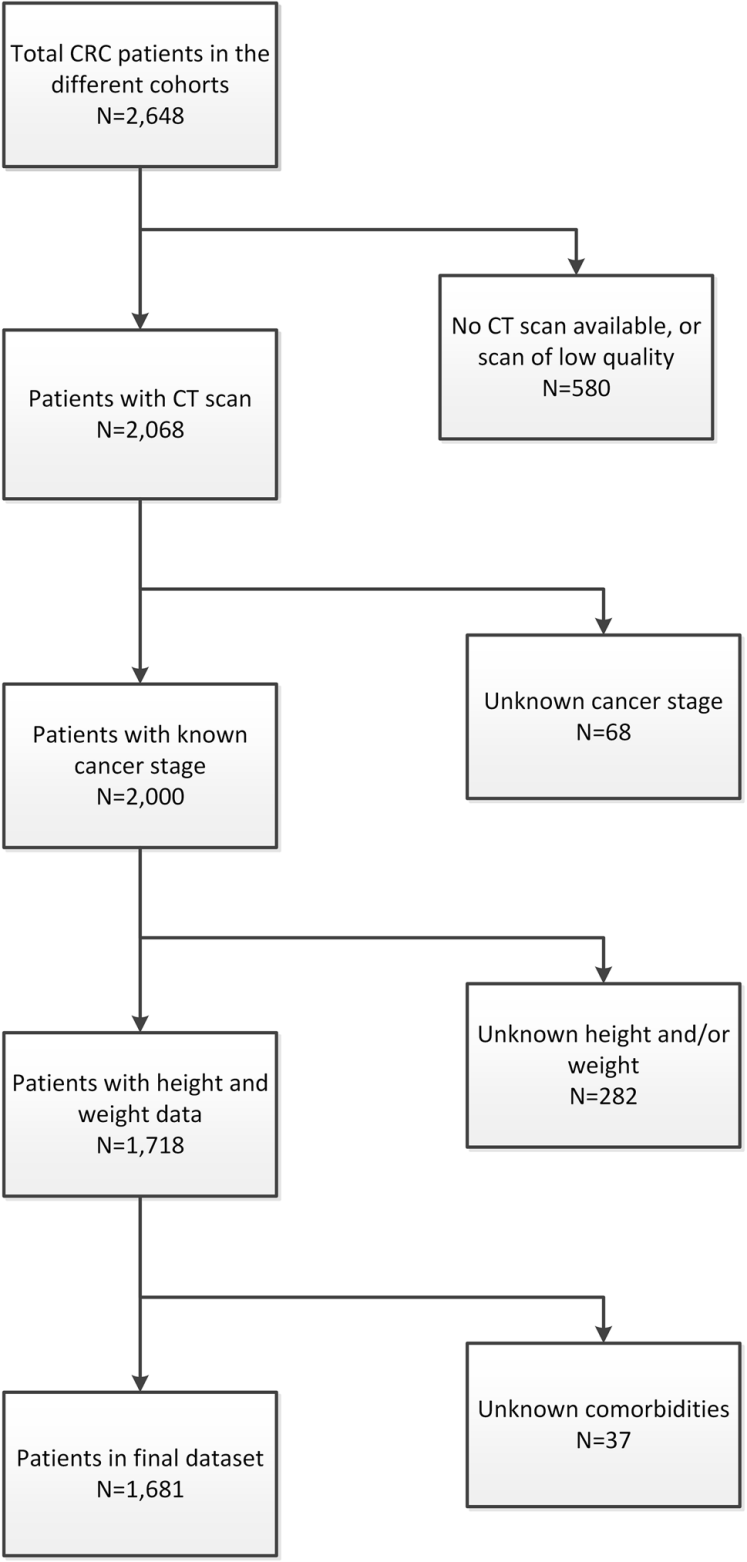
Flowchart of the study

Out of 2648 eligible patients, data from 580 patients had to be excluded because either no CT images were available, no pre-surgical CT image within 3 months of diagnosis was available, or the CT image was unusable due to low quality. An additional 68 patients were excluded because the stage of disease was unknown, for 282 patients data on height and/or weight were missing and for 37 patients comorbidity data were missing (Fig. 1). The final dataset consisted of 1681 patients. The average age of the 967 excluded patients was 68.5 ± 12.4 years; 43% were women; 61% had a tumor located in the colon; and of the excluded patients with stage of disease data available (79%), 26% had stage I disease, 38% stage II and 36% stage III CRC.

The average age of the study population was 67.7 ± 10.3 years and 41% were women; 67% of the patients had a tumor located in the colon (Table 1). The majority of these patients had a BMI between 25 and 29.9 kg/m^2^ (43%) or 20–24.9 kg/m^2^ (36%), while 17% had a BMI ≥ 30 kg/m^2^ and 4% a BMI < 20 kg/m^2^. In total, 414 patients (25%) died before January 31, 2017 and the median follow-up time was 48 months (range 0–119 months).

**Table 1:** Baseline characteristics in early-stage CRC patients with or without low skeletal muscle density

	Total population (*n* = 1681)	Skeletal muscle density	
		Low (*n* = 648) (39%)	Normal (*n* = 1033) (61%)
Demographic factors			
Age [years, mean (SD)]	67.7 (10.3)	73.2 (8.8)	64.3 (9.7)
Gender [*n* (%)]			
Men	999 (59)	372 (57)	627 (61)
Women	682 (41)	276 (43)	406 (39)
BMI [kg/m^2^, mean (SD)]			
BMI [kg/m^2^, *n* (%)]			
< 20	65 (4)	17 (3)	48 (5)
20–24.9	609 (36)	238 (37)	371 (36)
25–29.9	718 (43)	238 (37)	480 (47)
≥ 30	289 (17)	155 (24)	134 (13)
Clinical factors			
Cancer stage [*n* (%)]			
I	393 (23)	127 (20)	266 (26)
II	547 (33)	251 (39)	296 (29)
III	741 (44)	270 (42)	471 (46)
Tumor location [*n* (%)]^a^			
Colon	1123 (67)	465 (72)	658 (64)
Rectal	552 (33)	180 (28)	372 (36)
Number of comorbidities [*n* (%)]			
0–1	966 (58)	283 (44)	683 (66)
≥ 2	715 (43)	365 (56)	350 (34)
Treatment			
Radiotherapy [*n* (%)]^b^			
No	1136 (73)	462 (77)	674 (71)
Yes	419 (27)	140 (23)	279 (29)
Chemotherapy [*n* (%)]^c^			
No	1117 (72)	471 (78)	646 (68)
Yes	440 (28)	130 (22)	310 (32)
CT image analysis			
Skeletal muscle index [cm^2^/m^2^, mean (SD)]			
Men	51 (8)	48 (8)	53 (7)
Women	40 (6)	39 (6)	41 (6)
Muscle attenuation [HU, mean (SD)]			
Men	36 (8)	27 (5)	41 (5)
Women	32 (10)	23 (5)	39 (7)
Visceral adipose tissue [cm^2^, median (range)]	147 (0–535)	168 (1–535)	133 (0–495)
Subcutaneous adipose tissue [cm^2^, median (range)]	159 (0–713)	166 (0–713)	154 (1–596)
Total adipose tissue [cm^2^, median (range)]	341 (5–1070)	383 (35–1070)	316 (5–924)
Study [*n* (%)]			
Prospective	852 (51)	242 (37)	610 (59)
Registry-based	829 (49)	406 (63)	423 (41)
Follow-up time [months, median (range)]	48 (0–119)	48 (0–119)	48 (0–119)
Deceased patients [*n* (%)]	414 (25)	261 (40)	153 (15)

*BMI* body mass index, *SD* standard deviation, *HU* Hounsfield unit

^a^Data of six patients were missing

^b^Data of 126 patients were missing

^c^Data of 124 patients were missing

Thirty-nine percent of the patients had low skeletal muscle density (Table 1). Mean age and the percentage of women were higher in the low skeletal density group compared to the normal skeletal density group (73.2 ± 8.8 vs 64.3 ± 9.7 years; 43 vs 39%, respectively). Furthermore, the percentage of patients with a BMI ≥ 30 kg/m^2^ was higher in the low skeletal density group compared to the normal skeletal density group (24 vs 13%, respectively), while the percentage of patients with a BMI 25–29.9 kg/m^2^ was lower in the low skeletal muscle density group vs. the normal skeletal density group (37 vs 47%).

Data for CRC-specific mortality and disease-free survival were only available for participants of the COLON study. The average age of these patients was slightly lower than in the total study population: 65.6 ± 9.1 vs 67.7 ± 10.3 years (Table 2). Furthermore, the follow-up time was shorter (i.e., 37 vs. 48 months) and the percentage of deceased patients was lower (i.e., 11 vs. 25%). Other variables were comparable with the total population. Twenty-seven percent of the patients had low skeletal muscle density.

**Table 2:** Baseline characteristics of the participants from the COLON study

	COLON study (*n* = 715)	Skeletal muscle density	
		Low (*n* = 196) (27%)	Normal (*n* = 519) (73%)
Demographic factors			
Age [years, mean (SD)]	65.6 (9.1)	70.8 (7.8)	63.8 (8.9)
Gender [*n* (%)]			
Men	440 (62)	122 (62)	318 (61)
Women	275 (39)	71 (38)	201 (39)
BMI [kg/m^2^, *n* (%)]			
< 20	25 (4)	6 (3)	19 (4)
20–24.9	255 (36)	75 (38)	180 (35)
25–29.9	316 (44)	64 (33)	252 (49)
≥ 30	119 (17)	51 (26)	68 (13)
Clinical factors			
Cancer stage [*n* (%)]			
I	210 (29)	56 (29)	154 (30)
II	204 (29)	63 (32)	141 (27)
III	301 (42)	77 (39)	224 (43)
Tumor location [*n* (%)]			
Colon	483 (68)	136 (69)	247 (67)
Rectal	232 (32)	60 (31)	172 (33)
Number of comorbidities [*n* (%)]			
0–1	444 (62)	103 (53)	341 (66)
≥ 2	271 (38)	93 (47)	178 (34)
Follow-up time [months, median (range)]	37 (1–77)	38 (3–77)	36 (1–77)
Deceased patients [*n* (%)]	76 (11)	40 (24)	36 (7)

^a^Data of six patients missing

^b^Data of four patients missing

Low skeletal muscle density was significantly associated with higher mortality (low vs. normal skeletal muscle density: adjusted HR 1.91, 95% CI 1.53–2.38), Table 3. Number of comorbidities was identified as an effect modifier (*p* value for interaction = 0.02). In the stratified analysis, low skeletal muscle density was significantly associated with higher mortality in patients with zero or one comorbidities (low vs. normal skeletal muscle density: adjusted HR 1.67, 95% CI 1.20–2.31), yet this association was even stronger in the group of patients with two or more comorbidities (low vs. normal skeletal muscle density: adjusted HR 2.11, 95% CI 1.55–2.87).

**Table 3:** Association between skeletal muscle density and overall mortality

	No. of patients	No. of deaths *n* (%)	HR (95% CI)^a^
Skeletal muscle density, total population			
Normal	1033	153 (15)	REF
Low	648	261 (40)	1.91 (1.53–2.38)
Skeletal muscle density, 0–1 comorbidities			
Normal	683	93 (14)	REF
Low	283	88 (31)	1.67 (1.20–2.31)
Skeletal muscle density, ≥ 2 comorbidities			
Normal	350	60 (17)	REF
Low	365	173 (47)	2.11 (1.55–2.87)
Skeletal muscle density, HU (continuous)	1681	414 (25)	0.98 (0.96–0.99)

*HR* hazard ratio, *95% CI* 95% confidence interval, *REF* reference value, *HU* Hounsfield unit

^a^Adjusted for age, stage of disease, gender

Within the sub-population of the COLON study (*n* = 715), low skeletal muscle density was associated with higher overall mortality (low vs. normal skeletal muscle density: adjusted HR 2.15, 95% CI 1.32–3.50) and worse disease-free survival (low vs. normal skeletal muscle density: adjusted HR 1.68, 95% CI 1.14–2.47), but not significantly with colorectal cancer-specific disease (low vs. normal: adjusted HR 1.68, 95% CI 0.89–3.17) (Table 4).

**Table 4:** Association between skeletal muscle density and overall mortality, CRC-specific mortality and disease-free survival in a sub-population (*n* = 715)

	No. of patients	No. of deaths *n* (%)	HR (95% CI)^a^
Overall mortality			
Skeletal muscle density			
Normal	519	36 (7)	REF
Low	196	40 (20)	2.15 (1.32–3.50)
CRC-specific mortality			
Skeletal muscle density			
Normal	519	26 (5)	REF
Low	196	19 (10)	1.68 (0.89–3.17)
Disease-free survival			
Skeletal muscle density			
Normal	519	72 (14)	REF
Low	196	52 (27)	1.68 (1.14–2.47)

*HR* hazard ratio, *95% CI* 95% confidence interval, *REF* reference value

^a^Adjusted for age, stage of disease, gender

## Discussion

In our cohort of early-stage CRC patients, low skeletal muscle density was significantly associated with higher mortality. This association was strongest in patients with ≥ 2 comorbidities. Within a subset of the total study population, data on disease-free survival and cause of death were available, and low skeletal muscle density was associated with worse disease-free survival, but not statistically significantly with CRC-specific survival.

Our findings support the findings from Kroenke et al. (2018), who also found that low skeletal muscle density was associated with worse survival. Although speculative, our findings also underline the importance of using cut-off points for low skeletal muscle density that are defined in an appropriate population, as two other studies did not find associations between muscle density and survival (van Vugt et al. 2018; McSorley et al. 2017). The cut-off points we defined in our study population, are very comparable to the cut-offs defined in the study by Kroenke et al. (2018). The two studies that did not find associations, used cut-off levels for low skeletal muscle density that were defined in mixed group of cancer patients of various diagnoses, mostly with very poor prognosis (Martin et al. 2013).

In the present study, we did not find a statistically significant association between low skeletal muscle density and CRC-specific mortality. This might just be a matter of limited statistical power, as data on CRC-specific mortality were only available for the sub-population of participants enrolled in the COLON study. In the large study by Kroenke et al. (2018), an association was found with higher CRC-specific mortality. Further studies are needed to understand the mechanisms behind the association of skeletal muscle density and survival.

In the present study, the associations between low muscle density and both higher overall mortality and lower disease-free survival were independent of age, adipose tissue and BMI, suggesting that the patients with higher risk of dying were not just the older, obese CRC patients. It could be possible that patients with a high skeletal muscle density were more physically active in daily life. Studies investigating the effect of training and detraining (i.e., the effect of reduced physical training after a training program), and of strength and endurance training showed that those training programs increased skeletal muscle density (Lee et al. 2005; Taaffe et al. 2009; Poehlman et al. 2000), whereas detraining reduced skeletal muscle density (Taaffe et al. 2009). Very limited data are available on other determinants of skeletal muscle density, thus, whether other lifestyle factors like smoking and drinking are associated with decreased skeletal muscle density is still unknown.

We showed that the association of low muscle density and higher mortality was strongest among patients with multiple comorbidities. In a recent study (Xiao et al. 2018), an association between low skeletal muscle density and comorbidities was reported. Yet, our results suggests that lower skeletal muscle density is not simply a measure of worse health status, as among those patients with more comorbidities (thus with worst health status), the association was strongest. Potentially, the health status of those patients makes them extra vulnerable for the detrimental effects of fat infiltration in the muscle, but mechanistic studies are needed to explore this further.

This study has some limitations. First, data from patients without a CT image within 3 months of diagnosis had to be excluded. Since CT-diagnostic imaging was not common practice in clinical care of CRC in the Netherlands until 2008, a lot of scans were missing for patients diagnosed before 2008. Mean age, percentage of women, stage of disease and percentage of colon tumors were comparable between the included and excluded patient group. Therefore, we do not expect that excluding these patients affected our results. Second, due to the availability of cause of death and recurrence data, we could only perform the analyses for CRC-specific mortality and disease-free survival in the participants of the COLON study (*n* = 715).

This is the first large-scale European cohort that assessed the association between skeletal muscle density and survival with cut-off points relevant for/defined in our cohort of early-stage CRC patients. Other strengths are the large sample size, the long follow-up time and the inclusion of exclusively early-stage CRC patients.

In conclusion, low skeletal muscle density was significantly associated with higher overall mortality and lower disease-free survival in early-stage CRC patients. Future observational studies are needed to study which factors determine low skeletal muscle density. Thereafter, intervention studies should be performed to study whether intervening action taken on skeletal muscle density could improve prognosis of cancer patients, e.g., with nutritional interventions and/or exercise training.

## References

[CR1] Anderson DE, D’Agostino JM, Bruno AG, Demissie S, Kiel DP, Bouxsein ML (2013) Variations of CT-based trunk muscle attenuation by age, sex, and specific muscle. The J Gerontol Ser A Biol Sci Med Sci 68(3):317–323 22904095 10.1093/gerona/gls168 PMC3605905

[CR2] Antoun S, Lanoy E, Iacovelli R, Albiges-Sauvin L, Loriot Y, Merad-Taoufik M et al (2013) Skeletal muscle density predicts prognosis in patients with metastatic renal cell carcinoma treated with targeted therapies. Cancer 119(18):3377–3384 23801109 10.1002/cncr.28218

[CR3] Blauwhoff-Buskermolen S, Versteeg KS, de van der Schueren MA, Den Braver NR, Berkhof J, Langius JA et al (2016a) Loss of muscle mass during chemotherapy is predictive for poor survival of patients with metastatic colorectal cancer. J Clin Oncol 34(12):1339–1344 26903572 10.1200/JCO.2015.63.6043

[CR4] Blauwhoff-Buskermolen S, de van der Schueren MAE, Langius JAE, Verheul HMW (2016b) Reply to L.E. Daly et al. J Clin Oncol 34(31):3817 27480150 10.1200/JCO.2016.68.9364 PMC5477926

[CR5] Caan BJ, Meyerhardt JA, Kroenke CH, Alexeeff S, Xiao J, Weltzien E et al (2017) Explaining the obesity paradox: the association between body composition and colorectal cancer survival (C-SCANS Study). Cancer Epidemiol Prev Biomarkers 26(7):1008–1015 10.1158/1055-9965.EPI-17-0200 PMC5647152 28506965

[CR6] Daly LE, Ryan AM, Power DG (2016) Response to “Loss of muscle mass during chemotherapy is predictive for poor survival of patients with metastatic colorectal cancer”. J Clin Oncol 34(31):3816–3817 27480154 10.1200/JCO.2016.68.8010 PMC5477930

[CR7] Dijk DP, Bakens MJ, Coolsen MM, Rensen SS, Dam RM, Bours MJ et al (2017) Low skeletal muscle radiation attenuation and visceral adiposity are associated with overall survival and surgical site infections in patients with pancreatic cancer. J Cachexia Sarcopenia Muscle 8(2):317–326 27897432 10.1002/jcsm.12155 PMC5377384

[CR8] Ebadi M, Martin L, Ghosh S, Field CJ, Lehner R, Baracos VE et al (2017) Subcutaneous adiposity is an independent predictor of mortality in cancer patients. Br J Cancer 117(1):148–155 28588319 10.1038/bjc.2017.149 PMC5520211

[CR9] Fujiwara N, Nakagawa H, Kudo Y, Tateishi R, Taguri M, Watadani T et al (2015) Sarcopenia, intramuscular fat deposition, and visceral adiposity independently predict the outcomes of hepatocellular carcinoma. J Hepatol 63(1):131–140 25724366 10.1016/j.jhep.2015.02.031

[CR10] Goodpaster BH, Kelley DE, Thaete FL, He J, Ross R (2000) Skeletal muscle attenuation determined by computed tomography is associated with skeletal muscle lipid content. J Appl Physiol 89(1):104–110 10904041 10.1152/jappl.2000.89.1.104

[CR11] Goodpaster BH, Carlson CL, Visser M, Kelley DE, Scherzinger A, Harris TB et al (2001) Attenuation of skeletal muscle and strength in the elderly: the Health ABC study. J Appl Physiol 90(6):2157–2165 11356778 10.1152/jappl.2001.90.6.2157

[CR12] Kroenke CH, Prado CM, Meyerhardt JA, Weltzien EK, Xiao J, Cespedes Feliciano EM et al (2018) Muscle radiodensity and mortality in patients with colorectal cancer. Cancer 124(14):3008–3015 29797673 10.1002/cncr.31405 PMC6033621

[CR13] Lee S, Kuk JL, Davidson LE, Hudson R, Kilpatrick K, Graham TE et al (2005) Exercise without weight loss is an effective strategy for obesity reduction in obese individuals with and without type 2 diabetes. J Appl Physiol 99(3):1220–1225 15860689 10.1152/japplphysiol.00053.2005

[CR14] Martin L, Birdsell L, MacDonald N, Reiman T, Clandinin MT, McCargar LJ et al (2013) Cancer cachexia in the age of obesity: skeletal muscle depletion is a powerful prognostic factor, independent of Body Mass Index. J Clin Oncol 2012(45):722 10.1200/JCO.2012.45.2722 23530101

[CR15] McSorley ST, Black DH, Horgan PG, McMillan DC (2017) The relationship between tumour stage, systemic inflammation, body composition and survival in patients with colorectal cancer. Clin Nutr 37(4):1279–1285 28566220 10.1016/j.clnu.2017.05.017

[CR16] Mitsiopoulos N, Baumgartner R, Heymsfield S, Lyons W, Gallagher D, Ross R (1998) Cadaver validation of skeletal muscle measurement by magnetic resonance imaging and computerized tomography. J Appl Physiol 85(1):115–122 9655763 10.1152/jappl.1998.85.1.115

[CR17] Poehlman ET, Dvorak RV, DeNino WF, Brochu M, Ades PA (2000) Effects of resistance training and endurance training on insulin sensitivity in nonobese, young women: a controlled randomized trial 1. J Clin Endocrinol Metab 85(7):2463–2468 10902794 10.1210/jcem.85.7.6692

[CR18] Prado CM, Lieffers JR, McCargar LJ, Reiman T, Sawyer MB, Martin L et al (2008) Prevalence and clinical implications of sarcopenic obesity in patients with solid tumours of the respiratory and gastrointestinal tracts: a population-based study. Lancet Oncol 9(7):629–635 18539529 10.1016/S1470-2045(08)70153-0

[CR19] Rier HN, Jager A, Sleijfer S, van Rosmalen J, Kock MC, Levin M-D (2017) Low muscle attenuation is a prognostic factor for survival in metastatic breast cancer patients treated with first line palliative chemotherapy. Breast 31:9–15 27810702 10.1016/j.breast.2016.10.014

[CR20] Siegel RL, Miller KD, Fedewa SA, Ahnen DJ, Meester RG, Barzi A et al (2017) Colorectal Cancer Stat 2017 CA Cancer J Clin 67(3):177–193 10.3322/caac.21395 28248415

[CR21] Sjøblom B, Grønberg BH, Wentzel-Larsen T, Baracos VE, Hjermstad MJ, Aass N et al (2016) Skeletal muscle radiodensity is prognostic for survival in patients with advanced non-small cell lung cancer. Clin Nutr 35(6):1386–1393 27102408 10.1016/j.clnu.2016.03.010

[CR22] Taaffe DR, Henwood TR, Nalls MA, Walker DG, Lang TF, Harris TB (2009) Alterations in muscle attenuation following detraining and retrainingin resistance trained older adults. Gerontology 55(2):217 19060453 10.1159/000182084 PMC2756799

[CR23] van Roekel EH, Bours MJL, de Brouwer CPM, Ten Napel H, Sanduleanu S, Beets GL et al (2014) The applicability of the international classification of functioning, disability, and health to study lifestyle and quality of life of colorectal cancer survivors. Cancer Epidemiol Biomark Prev 23(7):1394–1405 10.1158/1055-9965.EPI-13-1144 24802740

[CR24] van Vugt JL, van den Braak RRC, Lalmahomed ZS, Vrijland WW, Dekker JW, Zimmerman DD et al (2018) Impact of low skeletal muscle mass and density on short and long-term outcome after resection of stage I-III colorectal cancer. Eur J Surg Oncol. 10.1016/j.ejso.2018.05.029 29914788 10.1016/j.ejso.2018.05.029

[CR25] Williams BA (2006) Finding optimal cutpoints for continuous covariates with binary and time-to-event outcomes. Rochester, MN, Mayo Foundation, Technical Report Series 79

[CR26] Winkels R, Heine-Broring R, Van Zutphen M, van Harten-Gerritsen S, Kok D, Van Duijnhoven F et al (2014) The COLON study: colorectal cancer: longitudinal, observational study on nutritional and lifestyle factors that may influence colorectal tumour recurrence, survival and quality of life. BMC Cancer 14(1):374 24886284 10.1186/1471-2407-14-374 PMC4046039

[CR27] Xiao J, Caan BJ, Weltzien E, Cespedes Feliciano EM, Kroenke CH, Meyerhardt JA et al (2018) Associations of pre-existing co-morbidities with skeletal muscle mass and radiodensity in patients with non-metastatic colorectal cancer. J Cachexia Sarcopenia Muscle. 10.1002/jcsm.12301 29675984 10.1002/jcsm.12301 PMC6104112

